# Integrated *in silico* Analyses of Regulatory and Metabolic Networks of *Synechococcus* sp. PCC 7002 Reveal Relationships between Gene Centrality and Essentiality

**DOI:** 10.3390/life5021127

**Published:** 2015-03-27

**Authors:** Hyun-Seob Song, Ryan S. McClure, Hans C. Bernstein, Christopher C. Overall, Eric A. Hill, Alexander S. Beliaev

**Affiliations:** Biological Sciences Division, Pacific Northwest National Laboratory, Richland, WA 99352, USA; E-Mails: ryan.mcclure@pnnl.gov (R.S.M.); hans.bernstein@pnnl.gov (H.C.B.); christopher.overall@pnnl.gov (C.C.O); eric.hill@pnnl.gov (E.A.H.)

**Keywords:** cyanobacteria, *Synechococcus* 7002, gene co-expression network, genome-scale metabolic network, node centrality, gene knock-out simulation, randomization test

## Abstract

Cyanobacteria dynamically relay environmental inputs to intracellular adaptations through a coordinated adjustment of photosynthetic efficiency and carbon processing rates. The output of such adaptations is reflected through changes in transcriptional patterns and metabolic flux distributions that ultimately define growth strategy. To address interrelationships between metabolism and regulation, we performed integrative analyses of metabolic and gene co-expression networks in a model cyanobacterium, *Synechococcus* sp. PCC 7002. Centrality analyses using the gene co-expression network identified a set of key genes, which were defined here as “topologically important.” Parallel *in silico* gene knock-out simulations, using the genome-scale metabolic network, classified what we termed as “functionally important” genes, deletion of which affected growth or metabolism. A strong positive correlation was observed between topologically and functionally important genes. Functionally important genes exhibited variable levels of topological centrality; however, the majority of topologically central genes were found to be functionally essential for growth. Subsequent functional enrichment analysis revealed that both functionally and topologically important genes in *Synechococcus* sp. PCC 7002 are predominantly associated with translation and energy metabolism, two cellular processes critical for growth. This research demonstrates how synergistic network-level analyses can be used for reconciliation of metabolic and gene expression data to uncover fundamental biological principles.

## 1. Introduction

The regulatory machinery of cyanobacteria, which evolved to provide ecophysiological advantages across a dynamic range of conditions, plays a major role in both short- and long-term adaptations. As regulatory controls dynamically couple the external inputs to intracellular adaptations, a coordinated adjustment of photosynthetic efficiency and carbon processing rates maximizes the organism’s energetic and metabolic efficiencies [1,2,3]. To identify the general principles governing the adaptive response of individual cyanobacterial species to environmental perturbations across different scales, from a single organism to community, a systems-level analysis requires integration of critical information on key genetic and metabolic mechanisms [4]. Such an integrative approach resolves several challenges that currently face the functional genomic analysis of model microbes and cyanobacteria, in particular. On one hand, it establishes functionality within the context of a genome-scale network and extends analysis beyond similarity comparison, which can be misleading or incorrect, due to the complicated evolutionary and structure-function relationships [5]. On the other hand, it allows for more facile application of comparative genomics tools and knowledge transfer from well-studied eubacterial species, which is limited, at best, due significant difference in the genome structure and regulon organization of cyanobacteria [6].

Genome-scale metabolic networks (GMNs) are becoming available for an increasing number of microorganisms through automated reconstruction pipelines and advanced manual curation [7,8]. GMNs provide strong stoichiometric constraints for metabolic reactions, by which the correlation between gene expression and metabolic fluxes is significantly improved [9]. Thus, GMNs have served as a useful tool for deepening our understanding of metabolism and the role of genes through the analysis of intracellular flux distributions and the evaluation of gene essentiality [10,11]. In contrast, gene co-expression network (GCN) collections are developing at a considerably slower pace due to the requirement of large data compendiums that examine the global gene expression patterns across a broad range of environmental conditions. These networks are built from several RNA-seq data sets and provide a map of gene relationships based on mutual information scores between gene expression levels. Related genes, or genes with important biological connections, can be identified through their correlation of expression across several RNA-seq data sets and an analysis of whether that correlation is statistically significant compared to background correlation between the gene of interest and every other gene in the transcriptome. To date, analyses of GCN are focused on network topology [12], functional characterization of correlated expression [13], and examination of network robustness [14]. The typical robustness analysis of GCNs investigates the effect of gene deletion on the properties of network structure, thus evaluating the topological significance of genes [15]. Despite the improved understanding of metabolism and gene expression patterns through those respective studies using metabolic and regulatory networks, there are still many fundamental questions that remain unanswered, specifically: (*i*) what is the relationship between topological and functional *importance* of genes in GCN and GMN; (*ii*) are topologically central genes in GCN also functionally essential in GMN; and (*iii*) if so, what are the characteristics of those genes? Previous studies in this area have addressed similar questions, however, using GMNs alone [16,17,18] or in combination with GMN-derived gene connection structures [19]. The present work is, to our knowledge, the first demonstration of integrative functional and topological analyses of multiple biological networks built independently from disparate data sources. 

Availability of well-established model systems, in conjunction with high-throughput “omics” data, provides the scale and resolution to perform integrative computational analyses of regulation and metabolism. In this study, we systematically compared transcript association patterns to metabolic capacity and interrogated the topological and functional importance of genes in a unicellular cyanobacterium *Synechococcus* sp. PCC 7002 (hereafter *Synechococcus* 7002). *Synechococcus* 7002 is a well-characterized microorganism capable of robust growth under a wide range of environmental conditions [20,21,22], in which adaptation to specific conditions is associated with distinct transcriptional patterns indicating tight regulation of metabolic and regulatory modules [23,24]. However, the degree by which transcriptional topology reflects essentiality of genes participating in central metabolic pathways remains unclear due to lack of approaches capable of relating large networked data sets in a quantitative fashion. In this study we present a novel computational framework to concurrently assess the centrality and functional importance of genes through integrative analysis of the GCN and GMN. This approach revealed that topologically central genes with high measures of networked centrality, measured over a broad range of distinct growth conditions, predominantly include those that are also functionally essential for cyanobacterial growth. 

## 2. Materials and Methods

### 2.1. Experimental Conditions and Measurements

Expression data for *Synechococcus* 7002, representing a total of 42 discrete growth conditions, were either generated from continuous cultures or sourced from previously reported studies, which examined growth of the organism under nutrient limitation, varying irradiance levels, extremes of cell density, temperature and salinity, as well as co-cultivation with a heterotrophic partner [23,24,25,26]. The continuous cultures of *Synechococcus* 7002, operated in chemostat or turbidostat modes, were grown on A+ media in photobioreactor at 30 °C with a dilution rate of 0.1 hr^−1^ as described previously [27]. In carbon-, nitrogen-, or light-limited chemostats, steady-state growth was limited by 7.7 mM NaHCO_3_, 0.9 mM NH_4_Cl, or 140 µE m^−2^ s^−1^, respectively. In turbidostat mode, *Synechococcus* 7002 was grown under six irradiance levels ranging from 33–758 µmol photons m^−2^ s^−1^ with 2% CO_2_ supplementation to gas (N_2_) sparged at 4 L min^−1^. Two other conditions consisted of a high light and high O_2_ (up to 60% v/v of sparge gas) adapted strain of *Synechococcus* 7002 grown under either 7.1% or 16.5% dissolved O_2_. Total RNA was extracted and sequenced as described previously [23,25] using a phenol chloroform approach and sequencing was performed using SOLiD 5500XL protocol with a read length of 50 bp [25] or with the SOLiD™ 3 or 3Plus protocol [23]. Gene expression levels were analyzed as reads per kilobase per million reads (RPKM) and all transcriptome data sets were compiled using the previously described Rockhopper software [28].

### 2.2. Metabolic Network Analysis of Wild-Type and Knock-Out Strains

Previously developed GMN of *Synechococcus* 7002 [29] was updated by: (i) changing incorrectly assigned gene-reaction association of r_AGPAT_SYN (1-acylglycerol 3-phosphate O-acyltransferase) from “SYNPCC7002_A0198” to “SYNPCC7002_A0918”; and (ii) separating glycogen from the biomass synthesis equation into the external metabolite pool. As the total number of genes contained in the resulting GMN was 706, we renamed the network model to *iSyp*706. 

**Figure 1:**
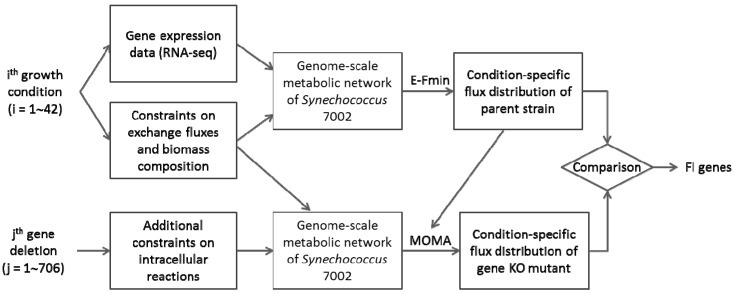
Analysis of the genome-scale metabolic network and identification of functionally important (FI) genes. The *in silico* knock-out simulations were conducted for all genes in the network (j = 1 to 706) under 42 expression-guided growth simulations (i = 1 to 42) to compare fluxes between the parent and knock-out networks as estimated via E-Fmin and MOMA, respectively.

*In silico* flux distributions were calculated for 42 growth conditions using the Expression-Guided Flux Minimization (E-Fmin) algorithm that incorporates RNA sequence data into a genome-scale metabolic network based on the flux minimization principle [30]. An advantage of E-Fmin is that it does not require the specification of a metabolic objective (to maximize or minimize), which is often difficult to reconcile with growth phase and condition. Instead, E-Fmin minimizes the sum of weighted flux magnitudes where the weight is formulated as a decreasing linear function of gene expression levels, ensuring that fluxes associated with genes expressed at low levels are significantly suppressed. *In silico* fluxes were compared through gene knock-out simulations using the Minimization of Metabolic Adjustment (MOMA) algorithm, which minimizes the Euclidean distance between two flux vectors of parent and mutant strains [31]. While the original formulation of MOMA is a constrained nonlinear optimization problem, we recasted it as a linear optimization problem for computational convenience. A gene was defined as functionally important (FI) if its inactivation produced changes in flux distributions as estimated by both E-Fmin and MOMA (Figure 1). 

### 2.3. Reconstruction of Gene Co-Expression Network (GCN)

Initial reconstruction of *Synechococcus* 7002 co-expression network were carried out at a genome-scale using all 3236 transcript-associated genes. To allow for cross-network comparisons, a reduced GCN containing only 706 reaction-associated genes included in the genome-scale metabolic network was generated. All network reconstructions were developed using Context Likelihood of Relatedness (CLR) program as previously described [14] and were based on the RPKM values from the 42 experimental conditions. A bootstrapping approach (500 iterations) was used to increase the robustness of the data. During each CLR iteration, two experimental conditions were randomly removed, and only those gene pairs (nodes) with a Z-score of 4.5 (4.5 standard deviations greater than the mean of all mutual information scores of that gene) in at least 375 of the runs were assigned edges between them. This served to remove weak edges and increased the quality of the resulting GCN. Groups of topologically and functionally important genes were subjected to functional enrichment analysis, which calculated the percentage of genes within a given functional category and identified those that are significantly higher than the percentage of genes of the same category in the entire genome with a *p*-value of >0.05 according to Fisher’s exact test.

### 2.4. Quantification of Gene Centrality

**Figure 2:**
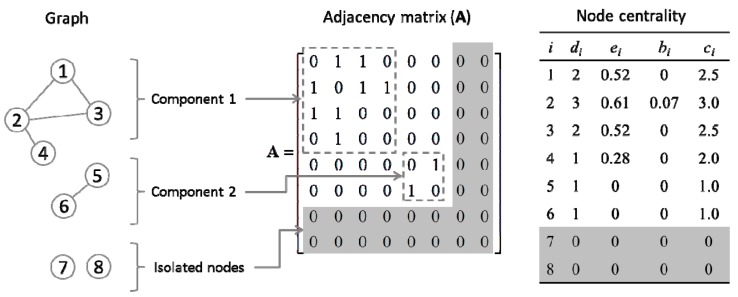
Graphical illustration of centrality concepts using a mock network. The nodes and edges, which represent genes and their co-expression relationships, respectively, are shown as an adjacency matrix (**A**). The graph is disconnected and the individual subgraphs are referred to as components. As centralities are zeros for isolated genes 7 and 8 (shaded in the table on the right), they can be optionally excluded from the adjacency matrix.

To evaluate the relative importance of genes in the GCN, we used four different types of centrality measures, including (i) degree; (ii) eigenvector; (iii) betweenness; and (iv) closeness (Figure 2). The degree ($d_{i}$), also known as connectivity, is the most elementary concept of centrality and denotes the number of links through which a node is directly connected to its neighbors. Degree centrality of node i is then expressed as:

(1) $$d_{i}=\sum_{j=1}^{n}A_{ij}$$

where $A_{ij}$ is the (i,j)^th^ element of the adjacency matrix **A**. The degree of centrality in Figure 2 ranges from 0 to 3.

A more refined connectivity term is eigenvector centrality ($e_{i}$), which accounts not only for the number of immediate neighbors, but also for their quality (*i.e.*, centrality). Thus the eigenvector centrality of node i is the summation of eigenvector centrality of its neighbors ($e_{j}$’s):

(2) $$e_{i}=\frac{1}{\lambda }\sum_{j=1}^{n}A_{ij}e_{j}$$

where λ is a constant. Equation (2) can be equivalently represented as a typical form of eigenvalue-eigenvector problem, *i.e.*, λe=Ae. In the example graph, while nodes 4 and 5 are connected to the same number of neighbors, node 4 is more central than node 5 due to the difference in the quality of neighbors. 

The term betweenness ($b_{i}$) defines centrality in terms of a number of shortest (or geodesic) paths passing through a given node in a network. Based on the assumption that information in a network flows through geodesic paths, betweenness quantitates the amount of information flowing through a given node (*n_i_*) and can be expressed as:

(3) $$b_{i}=\sum_{j,k\in N}\frac{\sigma \left(j,k\backslash i\right)}{\sigma \left(j,k\right)}$$

where N is the set of nodes in a network and σ(j,k) and σ(j,k\i) denote the number of geodesic paths between nodes j and k passing through node i. As shown in Figure 2, only node 2 has a nonzero value of betweenness. Note that node 1 is not between nodes 2 and 3 because 2-1-3 is not the shortest path between them. 

Finally, the term closeness (*c_i_*) indicates how close a given node is to the others; for instance, in a graph of n nodes, closeness centrality is measured by summing up the geodesic distances from a node to all other (n−1) nodes:

(4) $$c_{i}=\frac{1}{\sum_{j\in N}g_{ij}}$$

where $g_{ij}$ is the geodesic distance between nodes i and j. Note that closeness centrality defined in Equation (4) becomes zero for all nodes in a disconnected graph. To avoid this, we used the modified form of closeness [32] as given below:

(5) $$c_{i}=\sum_{j\in N}\frac{1}{g_{ij}}$$

As shown in Figure 2, nodes contained in the largest component (*i.e.*, component 1) have higher closeness values than those in the component 2. We calculated four centrality values of each gene in the GCN using the Matlab codes developed by Strategic Engineering Research Group (SERG) at MIT [33,34]. 

## 3. Results and Discussion 

### 3.1. Reconstruction and Topological Analysis of GCN

Biological networks often exhibit scale-free properties with inherent robustness to the random disruption of genes, but are vulnerable to targeted deletion of the most central gene nodes [35]. As a fundamental characteristic, the degree distribution of scale-free networks follows a power-law. This principle is shown in Supplementary Figure S1, which compares the 3236-gene (GCN_1_) and the 706 gene (GCN_2_) co-expression networks of *Synechococcus* 7002. Degree distributions of both networks appear as straight lines on log-log plots (Supplementary Figure S1a,b), implying that the developed co-expression networks of *Synechococcus* 7002 are robust and scale-free. While not apparent from the degree distributions, both GCNs are fragmented and composed of many disconnected subgraphs (or components). As a common feature of these two GCNs, the size of the largest component is significantly large relative to the other components. That is, the number of nodes of the first and second largest components of GCN_1_ (GCN_2_) was 985 (52) and 34 (10), respectively with all of the topologically central genes identified in our analysis clustering into the first largest components. As seen in the size distributions of GCN_1_ and GCN_2_ components (Supplementary Figure S1c,d), GCNs have a large portion of isolated genes, the number of which was 1842 in GCN_1_ and 495 in GCN_2_, respectively. As mentioned above (Material and Methods, Section 2.4), we removed these isolated genes for convenience of topological analysis and used (truncated) GCN_2_ throughout this work.

### 3.2. Centrality Analyses

**Figure 3:**
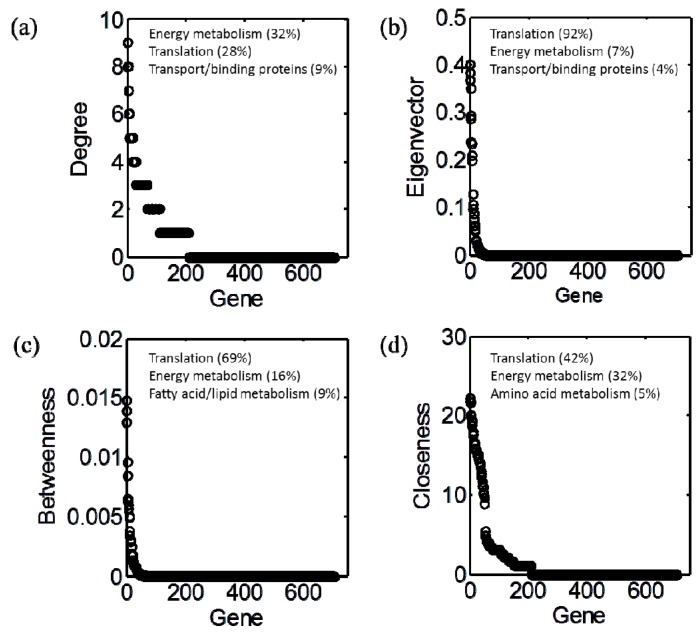
Distribution of gene centrality: (**a**) degree; (**b**) eigenvector; (**c**) betweenness; (**d**) closeness. In each panel, genes were sorted out in a descending order of the corresponding centrality measure.

The evaluation of gene centrality within GCN provided distinct distribution patterns across different centrality measures (Figure 3). Then, through functional enrichment analysis, we identified three major functional roles of genes, that are associated with different centrality measures. In all cases, translation and energy metabolism were the most dominant roles, though their relative dominance varied depending on the specific centrality measure. The largest fraction of energy metabolism genes that showed significant enrichment was those involved with either photosynthesis, electron transport, or oxidative phosphorylation, while smaller number encoded putative components of the carbon fixation machinery. In the case of translation, the vast majority of genes were part of either the large or small ribosomal subunit. Genes with high degree centrality were almost equally associated with energy metabolism and translation; eigenvector and betweenness centralities were predominantly associated with translation; closeness centralities were also closely associated with translation and energy metabolism at different levels. We defined topologically important (TI) genes as those within the top 50% measure of each centrality.

### 3.3. Identification of Functionally Important (FI) Genes

**Figure 4:**
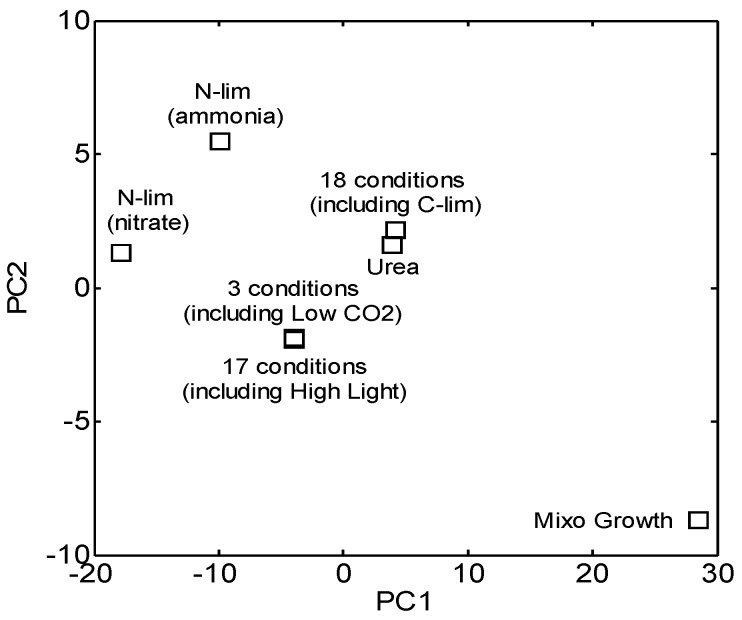
Principle component analysis (PCA) of estimated flux vectors in *Synechococcus* 7002 under 42 growth conditions. PCA identified only five different sets of conditions that lead to the difference in flux distribution. The first two principal components (PC1 and PC2) accounted for 99.4% of the variance.

While gene expression profiles varied with each growth condition, calculated flux distributions were relatively similar. Out of the 42 conditions tested, principle component analysis identified only 5 distinct flux distributions, indicating that the intracellular and exchange fluxes of *Synechococcus* 7002 are quite invariable (Figure 4, Supplementary Tables S1 and S2). These 5 sets of conditions are interpreted to have a sufficient level of “environmental variation” to profile the range adaptive responses which modulate *Synechococcus* 7002 metabolism. The limited variability of fluxes across the metabolic pathways of *Synechococcus* 7002 can be indicative of cyanobacterial growth robustness, which is somewhat intuitive, due to the relatively limited flexibility for energy and carbon acquisition pathways as compared to chemo- and photo-heterotrophs. 

The functional importance of genes was evaluated on the impact of metabolic flux distribution, which was predicted *in silico* by performing comprehensive single-gene knock-out simulations. Within the 706-gene network, we identified three different groups of genes with distinct functional importance. Deletion of any gene in Group 1 (362 genes) had no effect on *relative* flux distribution. This group was found to be enriched for genes involved in processes that are generally known to be non-essential for growth and/or facilitated by functionally redundant genes in *Synechococcus* 7002: *(i)* RNA polymerases (*p* < 10^−2^); *(ii)* folate synthesis (*p* < 10^−5^) and genes within the pentose phosphate pathway (*p* < 10^−3^). Deletion of any gene in Group 2 (55 genes) altered the relative flux distributions. Group 2 was functionally enriched for genes involved with processes that are known to have genetic redundancy and/or plasticity yet critical, condition specific roles for cyanobacterial growth: *(i)* glyoxylate/dicarboxylate metabolism (*p* < 10^−6^); *(ii)* pyruvate metabolism; *(iii)* oxidative phosphorylation (*p* < 10^−13^) and *(iv)* electron transport (*p* < 10^−3^). Any deletion of a gene in Group 3 (289 genes) resulted in no growth under at least one specific condition; 279 of the 289 Group 3 genes were lethal deletions under all conditions. Group 3 was functionally enriched for genes involved in critical growth processes such as: *(i)* photosynthesis (*p* < 10^−14^); *(ii)* carbon fixation (*p* < 0.05); *(iii)* tRNA synthesis (*p* < 10^−10^) and *(iv)* ribosome synthesis (*p* < 10^−23^). Computationally estimated flux distributions across conditions were provided in Supplementary Dataset S1. Based on this classification, we ranked functional importance of genes as Group 3 > Group 2 > Group 1 and referred to Group 2 and Group 3 as FI. It should be noted, that the results above could be affected to a degree depending on the accuracy of both E-Fmin and MOMA, methodologies employed for the *in silico* metabolic network analyses. While these methods have proven reliable in several case studies conducted in the original papers, direct validation using ^13^C-MFA data of *Synechococcus* 7002 has not yet been carried out and would be an important future direction for these studies.

### 3.4. Integration of Topological and Functional Analyses

**Figure 5:**
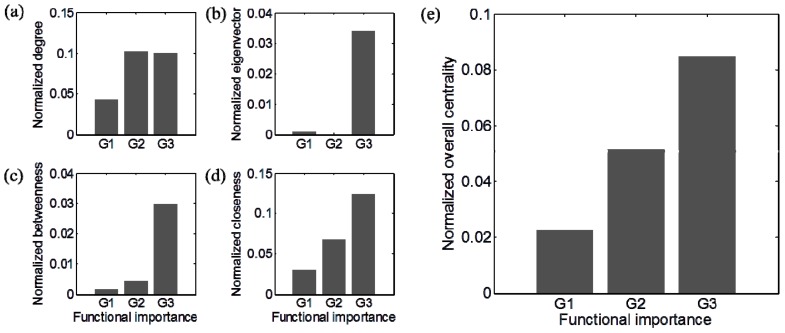
The relationship between topological and functional importance of genes: (**a**) degree; (**b**) eigenvector; (**c**) betweenness; (**d**) closeness; and (**e**) overall centrality. The overall centrality combines four individual centrality values. For normalization, each centrality measure was divided by its maximum value.

Comparative analysis of topologically and functionally distinct categories (*i.e.*, TI and FI genes) revealed positive correlation, albeit qualitative, between gene centralities and functional importance (Figure 5). Degree of centrality differentiated Groups 2 and 3 from Group 1 while, eigenvector and betweenness centralities separated Group 3 from Group 1 and Groups 2, showing their usefulness in identifying FI genes. Among four centrality measures considered in this work, however, closeness had the highest correlation with the functional importance of genes, clearly discerning all three groups. Such a proportional relationship was also observed when examining a combined centrality score. To assess the robustness of this analysis, we performed Monte Carlo simulations by randomly generating 5000 sets of genes of the same size as each group and comparing the centrality measures. From the difference in centrality measures between specific (Group 1 to Group 3) and randomly chosen groups, we could infer the same trends displayed in Figure 5 (Supplementary Figure S2). The profile patterns, corresponding to each measure of centrality, were considered with their top three biological roles (summarized in Figure 3) to identify the association of Group 3 and Group 2 with translation and energy metabolism, respectively. Association of Group 1 genes with specific biological functions was relatively weak in comparison to other groups. Details on gene functional enrichment of Group 1, Group 2, and Group 3 were provided in Supplementary Datasets S2, S3, and S4, respectively. 

**Figure 6:**
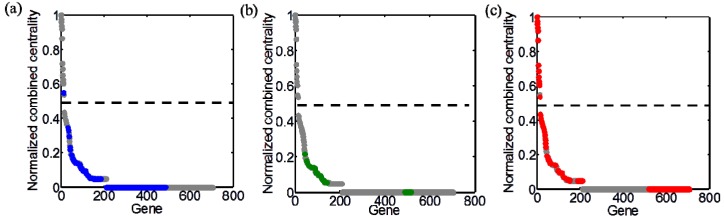
Distribution of genes with distinct functional importance along the combined centrality measure normalized by its maximum: (**a**) Group 1 (blue); (**b**) Group 2 (green); (**c**) Group 3 (red). Dashed line in each panel represents top 50% threshold.

**Figure 7:**
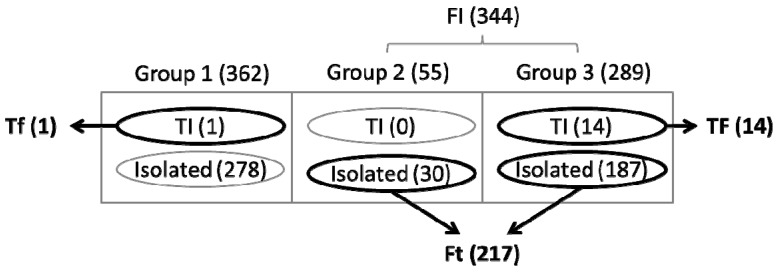
Classified gene groups by their functional and topological importance. 706 genes are divided into FI (*i.e.*, Group 2 and Group 3) and unimportant genes (Group 1). Each group contains topologically important (TI) genes and isolated genes. Three sets of particular interest include TI but not FI (Tf), FI but not TI (Ft), and intersection of TI and FI (TF) genes as highlighted in bold. The numbers in parentheses represent the number of genes belonging to each set.

The integration of topological and functional analyses provides a basis for the identification of the following three groups of genes: (i) TIs but not FI (here, denoted by Tf) genes; (ii) FI but not TI (Ft) genes; and (iii) intersection of TI and FI (TF) genes. As addressed earlier, we classified genes within the top 50% of the combined centrality scores as TI genes, while isolated genes (centrality values of 0) were classified as topologically unimportant (Figure 6). Most of TI genes belonged to the Group 3 (*i.e.*, essential genes) (thus, identified as TF), and as an exception, one gene belonged to the Group 1 (thus Tf). Interestingly, an appreciable portion of functionally essential genes (Group 1) were isolated nodes in the GCN as represented with zero centrality in Figure 6a. These topologically unimportant genes were also found in all other groups. The isolated genes among Group 2 and Group 3 thus belong to Ft. Figure 7 summaries the classification of 706 metabolic genes into different categories.

A relatively large number of *Synechococcus* 7002 genes were grouped within the Ft category (217), enabling a robust functional enrichment analysis. Several categories of genes were enriched when examining this list including those corresponding to processes that are required for production of biomass such as translation, and energy- and carbohydrate-metabolism (Supplementary Dataset S5). In addition, most of the Ft genes (~96%) were included in functionally enriched categories, confirming that the GMN model used in this study is well suited to represent the metabolism of *Synechococcus* 7002 across the 42 growth conditions considered. In fact, only a few genes on this list were involved in metabolic pathways that were not central to biomass production. All of the Ft genes were determined to be *isolated* nodes in the GCN implying that: (i) they are not critical for co-expression of other genes (because biomass synthesis belongs to the final process of metabolism); and (ii) the GCN is robust against these genes because the effect of their deletions does not propagate through the network). In contrast, the same classification criteria generated only one gene (*atp*A; syn7002_A0734) that belonged to the Tf group. The *in silico* GMN simulations suggested that the deletion of this gene had no effect on metabolite flux distributions, since *Synechococcus* 7002 has a homolog (*atp*A; syn7002_G0151) and that potentially function as ATPase in the absence of the syn7002_A0734 genes. Interestingly, genes categorized as TF were almost exclusively functional enriched for translation pathways (*i.e.*, 13 out of the 14 TF genes in total). These genes are topologically central, indicating that they play a key role in co-expression patterns of other genes. 

## 4. Concluding Remarks

Through integrated computational analyses of co-expression and metabolic networks in *Synechococcus* 7002, we interrogated biologically interesting questions related to the role of genes in cyanobacteria. The most fundamental result of this research was the interrelationship between the topological and functional importance of genes and that genes with high measures of centrality were predominantly found to be essential for growth and metabolism. Integrated network analysis revealed their overall correlation to be positive. The results from our analysis also suggest that genes with high centrality values in the co-expression network may indicate their important roles in metabolism. This work provides a bench mark foundation for integrated computational analyses of regulatory and metabolic processes and sets the stage for directed experiments to confirm if this approach will be able to provide predictive understandings of cyanobacterial growth, and other model organisms, across an even larger dynamic range of conditions. 

## Supplementary Files

Supplementary File 1

## References

[1] Bernstein H.C., Konopka A., Melnicki M.R., Hill E.A., Kucek L.A., Zhang S.Y., Shen G.Z., Bryant D.A., Beliaev A.S. (2014). Effect of mono- and dichromatic light quality on growth rates and photosynthetic performance of *synechococcus* sp pcc 7002. Front. Microbiol..

[2] Grossman A.R., Schaefer M.R., Chiang G.G., Collier J.L. (1993). The phycobilisome, a light-harvesting complex responsive to environmental-conditions. Microbiol. Rev..

[3] Grossman A.R., Schaefer M.R., Chiang G.G., Collier J.L. (2004). The responses of cyanobacteria to environmental conditions: Light and nutrients. The Molecular Biology of Cyanobacteria.

[4] Hernández-Prieto M.A., Semeniuk T.A., Futschik M.E. (2014). Toward a systems-level understanding of gene regulatory, protein interaction, and metabolic networks in cyanobacteria. Front. Genet..

[5] Strauss E.J., Falkow S. (1997). Microbial pathogenesis: Genomics and beyond. Science.

[6] Itoh T., Takemoto K., Mori H., Gojobori T. (1999). Evolutionary instability of operon structures disclosed by sequence comparisons of complete microbial genomes. Mol. Biol. Evol..

[7] Henry C.S., DeJongh M., Best A.A., Frybarger P.M., Linsay B., Stevens R.L. (2010). High-throughput generation, optimization and analysis of genome-scale metabolic models. Nat. Biotechnol..

[8] Thiele I., Palsson B.O. (2010). A protocol for generating a high-quality genome-scale metabolic reconstruction. Nat. Protocols.

[9] Moxley J.F., Jewett M.C., Antoniewicz M.R., Villas-Boas S.G., Alper H., Wheeler R.T., Tong L., Hinnebusch A.G., Ideker T., Nielsen J. (2009). Linking high-resolution metabolic flux phenotypes and transcriptional regulation in yeast modulated by the global regulator gcn4p. Proc. Natl. Acad. Sci. USA.

[10] Feist A.M., Palsson B.O. (2008). The growing scope of applications of genome-scale metabolic reconstructions using escherichia coli. Nat. Biotechnol..

[11] Oberhardt M.A., Palsson B.O., Papin J.A. (2009). Applications of genome-scale metabolic reconstructions. Mol. Syst. Biol..

[12] McDermott J.E., Taylor R.C., Yoon H.J., Heffron F. (2009). Bottlenecks and hubs in inferred networks are important for virulence in salmonella typhimurium. J. Comput. Biol..

[13] Ishchukov I., Wu Y., Van Puyvelde S., Vanderleyden J., Marchal K. (2014). Inferring the relation between transcriptional and posttranscriptional regulation from expression compendia. BMC Microbiol..

[14] Faith J.J., Hayete B., Thaden J.T., Mogno I., Wierzbowski J., Cottarel G., Kasif S., Collins J.J., Gardner T.S. (2007). Large-scale mapping and validation of escherichia coli transcriptional regulation from a compendium of expression profiles. PLoS Biol..

[15] Gibson S.M., Ficklin S.P., Isaacson S., Luo F., Feltus F.A., Smith M.C. (2013). Massive-scale gene co-expression network construction and robustness testing using random matrix theory. PLoS One.

[16] Wunderlich Z., Mimy L.A. (2006). Using the topology of metabolic networks to predict viability of mutant strains. Biophys. J..

[17] Palumbo M.C., Colosimo A., Giuliani A., Farina L. (2005). Functional essentiality from topology features in metabolic networks: A case study in yeast. Febs Lett..

[18] Mahadevan R., Palsson B.O. (2005). Properties of metabolic networks: Structure versus function. Biophys. J..

[19] Del Rio G., Koschutzki D., Coello G. (2009). How to identify essential genes from molecular networks?. BMC Syst. Biol..

[20] Liu H.B., Jing H.M., Wong T.H.C., Chen B.Z. (2014). Co-occurrence of phycocyanin- and phycoerythrin-rich *Synechococcus* in subtropical estuarine and coastal waters of Hong Kong. Environ. Microbiol. Rep..

[21] Wang K., Wommack K.E., Chen F. (2011). Abundance and distribution of *Synechococcus* spp. And cyanophages in the chesapeake bay. Appl. Environ. Microbiol..

[22] Van Baalen C. (1962). Studies on marine blue-green algae. Bot. Mar..

[23] Ludwig M., Bryant D.A. (2011). Transcription profiling of the model cyanobacterium *Synechococcus* sp. strain pcc 7002 by next-gen (SOLiD^TM^) sequencing of cDNA. Front. Microbiol..

[24] Ludwig M., Bryant D.A. (2012). *Synechococcus* sp strain pcc 7002 transcriptome: Acclimation to temperature, salinity, oxidative stress, and mixotrophic growth conditions. Front. Microbiol..

[25] Beliaev A.S., Romine M.F., Serres M., Bernstein H.C., Linggi B.E., Markillie L.M., Isern N.G., Chrisler W.B., Kucek L.A., Hill E.A. (2014). Inference of interactions in cyanobacterial-heterotrophic co-cultures via transcriptome sequencing. ISME J..

[26] Ludwig M., Bryant D.A. (2012). Acclimation of the global transcriptome of the cyanobacterium *Synechococcus* sp strain pcc 7002 to nutrient limitations and different nitrogen sources. Front. Microbiol..

[27] Melnicki M.R., Pinchuk G.E., Hill E.A., Kucek L.A., Stolyar S.M., Fredrickson J.K., Konopka A.E., Beliaev A.S. (2013). Feedback-controlled led photobioreactor for photophysiological studies of cyanobacteria. Bioresource Technol..

[28] McClure R., Balasubramanian D., Sun Y., Bobrovskyy M., Sumby P., Genco C.A., Vanderpool C.K., Tjaden B. (2013). Computational analysis of bacterial rna-seq data. Nucleic Acids Res..

[29] Hamilton J.J., Reed J.L. (2012). Identification of functional differences in metabolic networks using comparative genomics and constraint-based models. PLoS One.

[30] Song H.S., Reifman J., Wallqvist A. (2014). Prediction of metabolic flux distribution from gene expression data based on the flux minimization principle. PLoS One.

[31] Segre D., Vitkup D., Church G.M. (2002). Analysis of optimality in natural and perturbed metabolic networks. Proc. Natl. Acad. Sci. USA.

[32] Opsahl T., Agneessens F., Skvoretz J. (2010). Node centrality in weighted networks: Generalizing degree and shortest paths. Soc. Netw..

[33] Bounova G., de Weck O. (2012). Overview of metrics and their correlation patterns for multiple- metric topology analysis on heterogeneous graph ensembles. Phys. Rev. E.

[34] Octave Networks Toolbox First Release. https://zenodo.Org/record/10778.

[35] Albert R., Jeong H., Barabasi A.L. (2000). Error and attack tolerance of complex networks. Nature.

