# Supplementary material for: Integrated in silico Analyses of Regulatory and Metabolic Networks of Synechococcus sp. PCC 7002 Reveal Relationships between Gene Centrality and Essentiality
Source: Life (Basel). 2015 Mar 27;5(2):1127–40. doi: 10.3390/life5021127 (PMC4500133; doi:10.3390/life5021127)
Supplement: Supplementary File 1 [file life-05-01127-s001.zip › Supplementary tables and figures.pdf]

Supplementary Materials

Table S1. Summary of growth conditions with a short name given for each condition along with specifics of the growth conditions under analysis. RNA-seq data from several of these data sets has already been published and deposited into online databases with the references shown in the far right column.

#	Conditions	Specifics	Reference
1	C-lim	Synechococcus 7002 30 °C in continuous culture at a dilution rate of 0.1 hr^{-1}. A+ media was used with 7.7 mM $NaHCO_3$, 17 mM NH_4Cl and an incident light of 180 μE m^{-2} s^{-1} and was sparged with N_2 at a rate of 0.75 L/min.	This study
2	N-lim (ammonia)	Synechococcus 7002 30 °C in continuous culture at a dilution rate of 0.1 hr^{-1}. A+ media was used with 7.7 mM $NaHCO_3$, 0.9 mM NH_4Cl and an incident light of 180 μE m^{-2} s^{-1} and was sparged with N_2 at a rate of 0.75 L/min.	This study
3	L-lim	Synechococcus 7002 30 °C in continuous culture at a dilution rate of 0.1 hr^{-1}. A+ media was used with 7.7 mM $NaHCO_3$, 17 mM NH_4Cl and an incident light of 140 μE m^{-2} s^{-1} and was sparged with N_2 at a rate of 0.75 L/min.	This study
4	C-lim, HiLi/HiO2	Synechococcus 7002 OG1+LK1, high light / high O_2 chemostat	[1]
5	L-lim, LoLi/HiO2	Synechococcus 7002 OG1+LK1, low light / high O_2 chemostat	[1]
6	L-lim, LoLi/LoO2	Synechococcus 7002 OG1+LK1, low light / low O_2 chemostat	[1]
7	C-lim, HiLi/LoO2	Synechococcus 7002 OG1+LK1, high light / low O_2 chemostat	[1]
8	Shew Cocul, HiLi	Synechococcus 7002 30 °C in continuous culture chemostat with Shewanella W3-18. A+ media was used with 8 mM $NaHCO_3$ and spareged with 2% CO_2 in air with 1720 μmol photons m^{-2} s^{-1}	[1]
9	Shew Cocul, LoLi	Synechococcus 7002 30 °C in continuous culture chemostat with Shewanella W3-18. A+ media was used with 8 mM $NaHCO_3$ and spareged with 2% CO_2 in air with 640 μmol photons m^{-2} s^{-1}	[1]
10	Shew Cocul, lactate	Synechococcus 7002 30 °C in continuous culture chemostat with Shewanella W3-18. A+ media was used with 5mM lactate and spareged with 2% CO_2 in air with 1720 μmol photons m^{-2} s^{-1}	[1]
11	33umol photons	Synechococcus 7002 30 °C in continuous culture chemostat with A+ media and 17 mM NH_4Cl and irradiance of 33 μmol photons m^{-2} s^{-1}	This study
12	98umol photons	Synechococcus 7002 30 °C in continuous culture chemostat with A+ media and 17 mM NH_4Cl and irradiance of 98 μmol photons m^{-2} s^{-1}	This study
13	164umol photons	Synechococcus 7002 30 °C in continuous culture chemostat with A+ media and 17 mM NH_4Cl and irradiance of 164 μmol photons m^{-2} s^{-1}	This study
14	395umol photons	Synechococcus 7002 30 °C in continuous culture chemostat with A+ media and 17 mM NH_4Cl and irradiance of 395 μmol photons m^{-2} s^{-1}	This study

Table S1. *Cont.*

#	Conditions	Specifics	Reference
15	610umol photons	Synechococcus 7002 30 °C in continuous culture chemostat with A+ media and 17 mM NH_4Cl and irradiance of 610 µmol photons m^{-2} s^{-1}	This study
16	760umol photons	Synechococcus 7002 30 °C in continuous culture chemostat with A+ media and 17 mM NH4Cl and irradiance of 760 µmol photons m^{-2} s^{-1}	This study
17	Adapted Syn 7002 HiO2	Synechococcus 7002, high light/high O_2 adapted EH1—16.5% dissolved O_2	This study
18	Adapted Syn 7002 LoO2	Synechococcus 7002, high light/high O2 adapted EH1—7.1% dissolved O_2	This study
19	Standard	38 °C with continous illumination of 250 µE m^{-2} s^{-1} sparged with 1% CO_2 in air in 25 mL medium A with 1 mg/mL $NaNO_3$ (medium A+). Innoculated at $O.D._{730}$ 0.1, harvested at $O.D._{730}$ of 0.7	[2]
20	High Salt	Standard conditions but with 1.5 M NaCl and 40 mM KCL	[2]
21	Low Salt	Standard conditions but with 3 mM NaCl and 0.08 mM KCL	[2]
22	Mixo Growth	Standard growth conditions but with 10 mM glycerol	[2]
23	Ox Stress	30 minutes under standard conditions with 5uM methyl viologen	[2]
24	42 Deg	Standard conditions but at 42 °C for 1 h	[2]
25	30 Deg	Standard growth but at 30 °C	[2]
26	22 Deg	Standard growth but at 22 °C	[2]
27	Urea	Standard growth in medium A (not +) with 25 mM HEPES (no Tris-HCL), 1 µM $NiSO_4$ and 10 mM urea, grown to final OD of 0.7 before harvest	[3]
28	Ammonia	Standard growth in medium A (not +) with 25 mM HEPES (no Tris-HCL), 1 µM $NiSO_4$ and 10 mM NH_4Cl, grown to final OD of 0.7 before harvest	[3]
29	Nitrate	Standard growth in medium A (not +) with 25 mM HEPES (no Tris-HCL), 1 µM $NiSO_4$ and 12 mM $NaNO_3$, grown to final OD of 0.7 before harvest	[3]
30	Fe-lim	At standard conditions and an OD of 0.35 a final concentration of 720 µM deferoxamine mesylate B was added to cultures and harvest took place at an OD of 0.7	[3]
31	P-lim	Growth under standard conditions until OD reached 0.6–0.7, cells were then centrifuged and washed twice in medium A+ with no phosphate and allowed to grow from OD of 0.25 to 0.7 before harvest	[3]

Table S1. *Cont.*

#	Conditions	Specifics	Reference
32	S-lim	Growth under standard conditions until OD reached 0.6–0.7, cells were then centrifuged and washed twice in medium A+ with $MgCl_2$ instead of $MgSO_4$ and allowed to grow from OD of 0.35 to 0.7 before harvest	[3]
33	N-lim (nitrate)	Growth under standard conditions until OD reached 0.6-0.7, cells were then centrifuged and washed twice in medium A+ with no nitrate and allowed to grow from OD of 0.35 to 0.7 before harvest	[3]
34	Low CO_2	Standard conditions but sparged with air (0.035% CO_2)	[3]
35	Low O_2	Standard conditions but sparged with 1% CO_2 in N_2	[3]
36	O.D. 0.4	Starting OD of 0.05–0.1 under standard conditions and harvested with OD reached 0.4	[4]
37	O.D. 1.0	Starting OD of 0.05–0.1 under standard conditions and harvested with OD reached 1.0	[4]
38	O.D. 3.0	Starting OD of 0.05–0.1 under standard conditions and harvested with OD reached 3.0	[4]
39	O.D. 5.0	Starting OD of 0.05–0.1 under standard conditions and harvested with OD reached 5.0	[4]
40	High Light	Growth under standard conditions to OD of 0.7 and 1 hour of 900 μmol photons m^{-2} s^{-1}	[4]
41	Dark Anoxic	Growth under standard conditions to OD of 0.7 and 1 hour of dark and sparging with 1% CO_2 in N_2	[4]
42	Dark Oxic	Growth under standard conditions to OD of 0.7 and 1 hour of dark	[4]

Table S2. Classification of 42 growth conditions into different sets based on the similarity of *in silico* flux distributions as identified by principle component analysis. No substantial differences in flux distribution were observed between Sets 3a and 3b, and between Sets 4a and 4b. Full description of each growth condition is available in Table S1.

Set	Growth conditions
**1**	N-lim (ammonia)
**2**	N-lim (nitrate)
**3**	(**3a**) C-lim; L-lim; C-lim, HiLi/HiO2; L-lim, LoLi/LoO2; L-lim, LoLi/HiO2; C-lim, HiLi/LoO2; Shew Cocul, HiLi; Shew Cocul, LoLi; Shew Cocul, lactate; 33 µmol photons; 98 µmol photons; 164 µmol photons; 395 µmol photons; 610 µmol photons; 760 µmol photons; Adapted Syn 7002 HiO2; Adapted Syn 7002 LoO2; Ammonia
	(**3b**) Urea
**4**	(**4a**) Standard; High Salt; Low Salt; Ox Stress; 42 Deg; 30 Deg; 22 Deg; Nitrate; Fe-lim; P-lim; S-lim; Low CO_2; Low O_2; O.D. 0.4; O.D. 1.0; O.D. 3.0; O.D. 5.0; High Light
	(**4b**) Low CO_2; Dark Anoxic; Dark Oxic
**5**	Mixo Growth

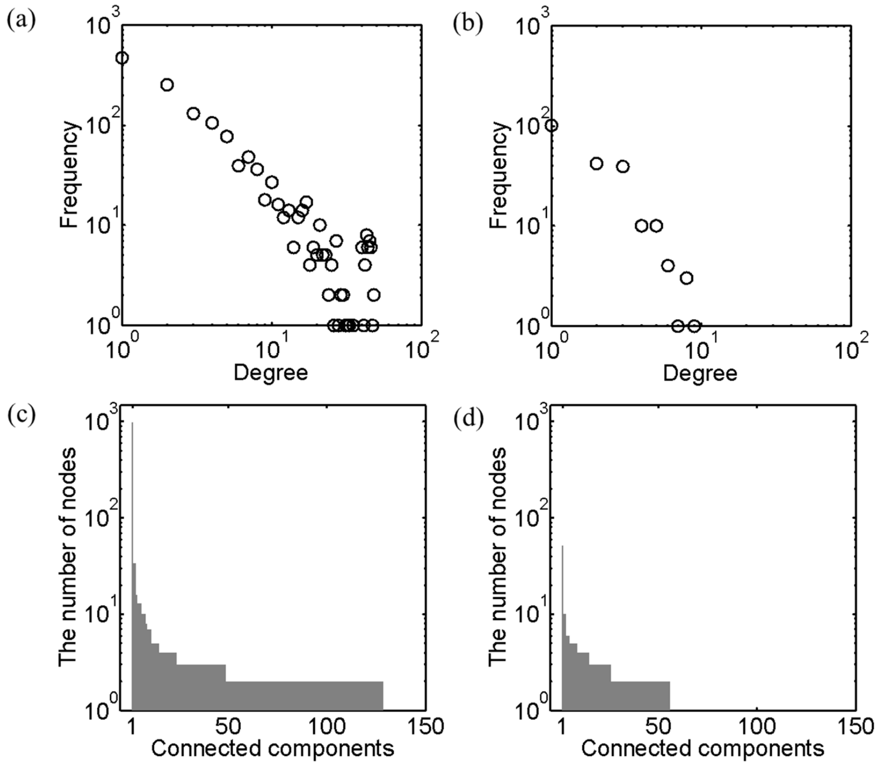

Figure S1. Degree (**a,b**) and component size (**c,d**) distributions in two GCNs constructed from the whole 3236 genes (GCN_1) and 706 metabolic genes (GCN_2).

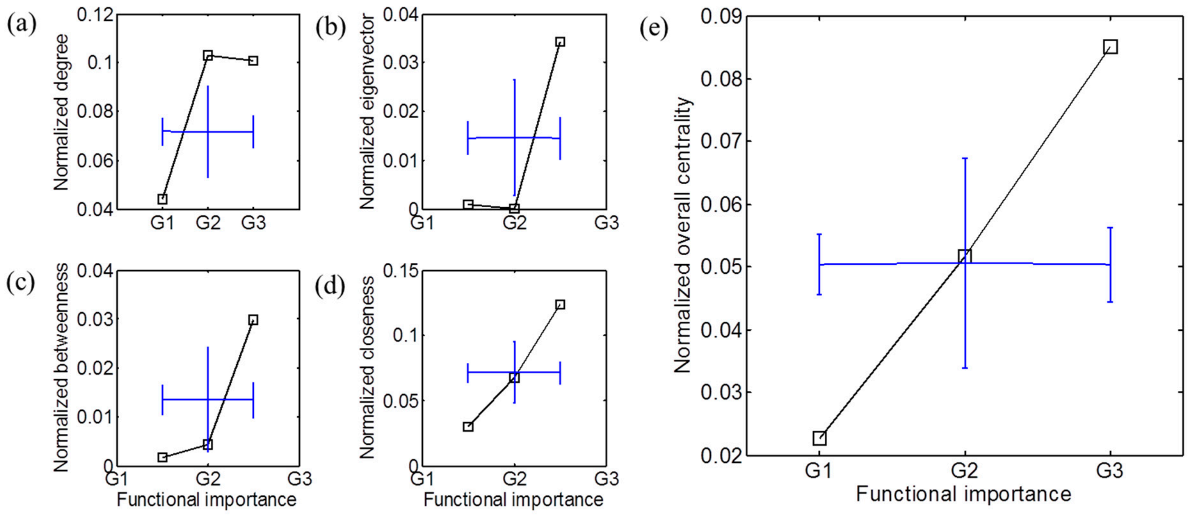

Figure S2. Comparison of the centrality values between specifically defined groups (Group 1 to Group 3) (black) and randomly chosen groups (blue). The vertical bars along the blue lines represent standard deviation of the average centrality values among 5000 randomly selected groups.

References

1. Beliaev, A.S.; Romine, M.F.; Serres, M.; Bernstein, H.C.; Linggi, B.E.; Markillie, L.M.; Isern, N.G.; Chrisler, W.B.; Kucek, L.A.; Hill, E.A.; *et al.* Inference of interactions in cyanobacterial-heterotrophic co-cultures via transcriptome sequencing. *ISME J.* **2014**, *8*, 2243–2255.
2. Ludwig, M.; Bryant, D.A. *Synechococcus* sp. strain PCC 7002 transcriptome: Acclimation to temperature, salinity, oxidative stress, and mixotrophic growth conditions. *Front Microbiol.* **2012**, *3*, doi:10.3389/fmicb.2012.00354.
3. Ludwig, M.; Bryant, D.A. Acclimation of the global transcriptome of the cyanobacterium *synechococcus* sp strain PCC 7002 to nutrient limitations and different nitrogen sources. *Front Microbiol.* **2012**, *3*, doi:10.3389/fmicb.2012.00145.
4. Ludwig, M.; Bryant, D.A. Transcription profiling of the model cyanobacterium *synechococcus* sp. strain PCC 7002 by next-gen (SOLiD^{TM}) sequencing of cDNA. *Front Microbiol.* **2011**, *2*, doi:10.3389/fmicb.2011.00041.
